# DNR Code Status Is Not Associated with Under-Utilization of Inpatient Transthoracic Echocardiograms

**DOI:** 10.3390/jcdd8090112

**Published:** 2021-09-15

**Authors:** Adarsh Katamreddy, Aaron J. Wengrofsky, Weijia Li, Cynthia C. Taub

**Affiliations:** 1 Internal Medicine, Jacobi Medical Center, Bronx, NY 10461, USA; katamrea@nychhc.org (A.K.); wli15@montefiore.org (W.L.); 2 Internal Medicine, Montefiore Medical Center, Bronx, NY 10467, USA; awengrofsk@montefiore.org; 3 Cardiology, Dartmouth-Hitchcock Medical Center, Lebanon, NH 03766, USA

**Keywords:** do not resuscitate, transthoracic echocardiogram, code status

## Abstract

In the strictest sense, do-not-resuscitate (DNR) status means that cardiopulmonary resuscitation should not be performed after death has occurred; all other medical interventions in line with a patient’s goals of care should be implemented. The use of transthoracic echocardiography (TTE) in patients with DNR status is unknown. Therefore, we aim to evaluate the utilization of TTE among patients with DNR status using this retrospective data analysis. A total of 16,546 patient admissions were included in the final study. A total of 4370 (26.4%) of the patients had a TTE during hospitalization; among full code patients, 3976 (25.7%) underwent TTE, whereas TTEs were performed in 394 (37.4%) of DNR patients. On univariate logistic regression analysis, full code status had OR (95% confidence interval, CI) 0.57 (0.51–0.66), *p* < 0.01 compared with DNR status for the performance of inpatient TTE. In the final multivariate model adjusted for age, sex, race, and clinical comorbidities, the full code patients had OR (95% CI) 0.91 (0.79–1.05), *p* = 0.22 compared with DNR patients for the performance of inpatient TTE. DNR status is not associated with a decrease in inpatient transthoracic echocardiography performance.

## 1. Introduction

In the strictest sense, do-not-resuscitate (DNR) status means that cardiopulmonary resuscitation should not be performed after death has occurred; all other medical interventions in line with a patient’s goals of care should be implemented. However, studies have shown that internal medicine resident physicians assume patients who do not wish to be resuscitated would prefer not to receive other treatments [1]. The use of transthoracic echocardiography (TTE) in patients with DNR status is unknown. This commonly performed noninvasive diagnostic modality provides valuable information regarding the structure and function of the heart [2]. With healthcare costs continuing to increase in the United States, appropriate use of diagnostic tests is essential [3]. Furthermore, inappropriate use of diagnostic modalities can be harmful rather than beneficial for patients. Therefore, we aim to evaluate the utilization of TTE among patients with DNR status. We hypothesized that patients with a DNR status undergo a similar number of TTE evaluations compared to full code patients.

## 2. Materials and Methods

Our study is a retrospective, cross-sectional, observational study. All patients over 18 years of age admitted to the three academic hospitals within Montefiore Medical Center between 15 March 2019 and 31 May 2019 were included in the study. Clinical data were acquired from the EPIC electronic medical record system. The study was approved by the Albert Einstein College of Medicine Institutional Review Board. Age at admission, gender, self-reported race, and code status at the time of TTE were recorded. Clinical risk factors including history of diabetes, hypertension, heart failure, cerebrovascular accident, liver disease, kidney disease, malignancy, and peripheral vascular disease were collected using ICD-10 codes and mapped diagnosis codes. ICD-10 codes used were the following: diabetes E08-E13, hypertension I10-I16, heart failure I50, cerebrovascular accident G45.0, G45.1, G45.2, G45.8, G45.9, G46.0, G46.1, G46.2, I60, I61, I62, and I63, liver disease K70-K77, kidney disease N17-19, peripheral vascular disease I73. The Elixhauser comorbidity index is a tool to adjust for baseline risk factors associated with in-hospital mortality in administrative data [4]. Electronic medical records were manually reviewed for patients with DNR code status at the time of transthoracic echocardiogram to assess if they were comfort care. Patients with comfort care status at the time of the TTE were excluded from the analysis. A univariate logistic regression with the code status, clinical risk factors, demographic variables, and confounders as covariates was carried out with the performance of transthoracic echocardiography as the outcome. All statistically significant variables were included in binary multivariate logistic regression analysis. A two-tailed *p* value less than 0.05 was considered statistically significant.

## 3. Results

The final analysis included a total of 16,546 patient admissions. Overall, 34.1% of patients identified as African American, 13.5% were Caucasian, 40.7% were Hispanic, and 11.7% identified as other. Additionally, 15,492 were full code and 1054 were DNR at the time of transthoracic echocardiography examination. The full code patients were younger (median [interquartile range] 60 [45–72] years) than DNR patients (80 [69–88.5] years). A total of 4370 (26.4%) of the patients had a TTE during hospitalization; 3976 (25.7%) were in full code patients and 394 (37.4%) were among DNR patients (Table 1).

On univariate logistic regression analysis, full code status had OR (95% confidence interval, CI) 0.57 (0.51–0.66), *p* < 0.01 compared with DNR status for the performance of inpatient TTE. In the final multivariate model adjusted for age, sex, race, and clinical comorbidities, the full code patients had OR (95% CI) 0.91 (0.79–1.05), *p* = 0.22 compared with DNR patients for the performance of inpatient TTE (Table 2). Further, Hispanic patients had OR (95% CI) 0.82 (0.76–0.89), *p* < 0.01 compared to African Americans. A total of 10 TTE examinations were performed in patients with comfort care status. These patients were excluded from the final analysis. 

## 4. Discussion

Our study found that there was no statistically significant difference in the performance of inpatient transthoracic echocardiograms among the patients with DNR code status compared with full code in a multiethnic cohort. This finding is in line with appropriate care provided to DNR patients. While studies have indicated a difference in the performance of invasive diagnostic tests such as bronchoscopy, data regarding TTE was not available. Our study is the first study looking at TTE performance among DNR and full code patients.

### 4.1. Predictors of Inpatient Transthoracic Echocardiogram Performance

Hispanic patients had lower odds for inpatient TTE performance compared to African Americans. The “Hispanic paradox”, when Hispanic patients have a lower incidence of cardiovascular disease than African Americans and Caucasians with similar risk factors, is a possible explanation for this finding [5]. Patients with a history of myocardial infarction (MI) and stroke had increased odds for the performance of inpatient TTE consistent with an increased risk of cardiac dysfunction associated with these risk factors. While peripheral artery disease was associated with increased odds for inpatient TTE on univariate analysis, this association was lost in the multivariate analysis due to possible confounding with other risk factors such as MI, stroke, etc. Another interesting finding was that a history of cancer was not associated with increased odds of inpatient TTE performance. We analyzed only inpatient examinations; we did not explore outpatient TTE. Therefore, this finding needs to be examined in future studies with inpatient and outpatient TTEs.

### 4.2. Strengths and Limitations

Although our study showed that patients with DNR status are receiving appropriate care at our center, it is limited due to the single-center, retrospective nature of the study. However, the diverse patient population included in our study makes the results more generalizable. Our study and findings shine a light on the importance of not withholding necessary cardiac diagnostic tests and treatments in patients with DNR status. Further, patients with comfort-only measures should not receive TTE as this is unlikely to change the overall management and may even be harmful by prompting further downstream testing.

### 4.3. Clinical Implications

This study found that clinicians in our institution did not conflate DNR with comfort care, an appropriate distinction. It is essential that patients receive care that is deemed medically appropriate and in line with a patient’s goals of care, not code status.

## 5. Conclusions

DNR status is not associated with a decrease in inpatient transthoracic echocardiography performance. 

## Figures and Tables

**Table 1:** Patient characteristics.

*n*	Overall 16,546	Full Code 15,492	DNR 1054	*p*-Value
Age (years), median [Q1, Q3]	61.0 [47.0, 74.0]	60.0 [45.0, 72.0]	80.0 [69.0, 88.5]	<0.001
Male, *n* (%)	7014 (42.4)	6569 (42.4)	445 (42.2)	0.933
Race				
African American, *n* (%)	5637 (34.1)	5304 (34.2)	333 (31.6)	<0.001
Caucasian, *n* (%)	2238 (13.5)	1996 (12.9)	242 (23.0)	
Hispanic, *n* (%)	6734 (40.7)	6376 (41.2)	358 (34.0)	
Other, *n* (%)	1937 (11.7)	1816 (11.7)	121 (11.5)	
Comorbidity				
Stroke, *n* (%)	1062 (6.4)	945 (6.1)	117 (11.1)	<0.001
Myocardial infarction, *n* (%)	1350 (8.2)	1211 (7.8)	139 (13.2)	<0.001
Peripheral artery disease, *n* (%)	565 (3.4)	516 (3.3)	49 (4.6)	0.028
Heart failure, *n* (%)	5672 (34.3)	5280 (34.1)	392 (37.2)	<0.001
Diabetes mellitus, *n* (%)	5672 (34.3)	5280 (34.1)	392 (37.2)	0.043
Liver disease, *n* (%)	259 (1.6)	238 (1.5)	21 (2.0)	0.305
Hypertension, *n* (%)	7970 (48.2)	7435 (48.0)	535 (50.8)	0.088
Malignancy, *n* (%)	1687 (10.2)	1502 (9.7)	185 (17.6)	<0.001
Kidney, *n* (%)	4223 (25.5)	3791 (24.5)	432 (41.0)	<0.001
Elixhauser comorbidity index, median [Q1, Q3]	4.0 [0.0, 14.0]	4.0 [0.0, 14.0]	4.0 [0.0, 14.0]	0.688
Transthoracic echocardiogram, *n* (%)	4370 (26.4)	3976 (25.7)	394 (37.4)	<0.001

**Table 2:** Univariate and multivariate logistic regression for performing transthoracic echocardiography.

	Univariate Models		Multivariate Model ^i^	
	OR (95% CI) ^g^	*p*-Value ^h^	OR (95% CI) ^g^	*p*-Value ^h^
Age ^a^	1.02 (1.02–1.02)	< 0.01	1.02 (1.02–1.02)	<0.01
Sex ^b^	1.36 (1.27–1.46)	< 0.01	1.29 (1.19–1.39)	<0.01
Race (Caucasian) ^c^	1.10 (0.99–1.23)	0.06	0.96 (0.86–1.08)	0.51
Race (Hispanic) ^d^	0.79 (0.73–0.85)	< 0.01	0.82 (0.76–0.89)	<0.01
Race (Other) ^e^	0.78 (0.69–0.87)	< 0.01	0.88 (0.78–0.99)	0.04
Comorbidities ^f^				
Stroke	2.73 (2.40–3.09)	< 0.01	2.19 (1.92–2.50)	<0.01
Myocardial infarction	2.78 (2.48–3.11)	< 0.01	1.81 (1.60–2.05)	<0.01
Peripheral artery disease	1.27 (1.06–1.52)	< 0.01	0.64 (0.52–0.78)	<0.01
Heart failure	3.29 (3.04–3.55)	< 0.01	2.51 (2.32–2.75)	<0.01
Diabetes mellitus	1.66 (1.54–1.78)	< 0.01	1.14 (1.06–1.24)	<0.01
Hypertension	0.96 (0.90–1.03)	0.34		
Liver disease	1.12 (0.85–1.46)	0.31		
Malignancy	0.83 (0.73–0.93)	< 0.01	0.70 (0.62–0.79)	<0.01
Kidney disease	1.57 (1.45–1.69)	< 0.01	0.87 (0.80–0.95)	<0.01
Elixhauser comorbidity score	1.00 (0.99–1.00)	0.92		
Full code ^j^	0.57 (0.51–0.66)	< 0.01	0.91 (0.79–1.05)	0.22

^a^ Age in years. ^b^ Male compared to female. ^c^ Caucasian compared to African American. ^d^ Hispanic compared to African American. ^e^ Other race compared to African American. ^f^ Current or past medical history. ^g^ Odds ratio (95% confidence interval). ^h^ *p* value for Wald χ^2^ test of β coefficient in the logistic regression model. ^i^ The multivariate model adjusts for age, sex, race, stroke, myocardial infarction, peripheral artery disease, heart failure, diabetes mellitus, malignancy, kidney disease, and code status. ^j^ Full code compared to DNR.

## Data Availability

Data will be provided on reasonable request.
